# A novel synonymous *ABCA3* variant identified in a Chinese family with lethal neonatal respiratory failure

**DOI:** 10.1186/s12920-021-01098-4

**Published:** 2021-10-29

**Authors:** Weifeng Zhang, Zhiyong Liu, Yiming Lin, Ruiquan Wang, Jinglin Xu, Ying He, Fengfeng Zhang, Lianqiang Wu, Dongmei Chen

**Affiliations:** 1 Department of Neonatal Intensive Care Unit, Quanzhou Maternity and Children’s Hospital, 700 Fengze Street, Quanzhou, 362000 Fujian Province China; 2 Neonatal Disease Screening Center, Quanzhou Maternity and Children’s Hospital, 700 Fengze Street, Quanzhou, 362000 Fujian Province China; 3 Xiamen Genokon Medical Technology Co., Ltd., Xiamen, 361000 Fujian Province China

**Keywords:** *ABCA3* gene, Synonymous variant, Cryptic splice site, Pulmonary surfactant, Lethal neonatal respiratory failure

## Abstract

**Background:**

Lethal respiratory failure is primarily caused by a deficiency of pulmonary surfactant, and is the main cause of neonatal death among preterm infants. Pulmonary surfactant metabolism dysfunction caused by variants in the *ABCA3* gene is a rare disease with very poor prognosis. Currently, the mechanisms associated with some *ABCA3* variants have been determined, including protein mistrafficking and impaired phospholipid transport. However, some novel variants and their underlying pathogenesis has not been fully elucidated yet. In this study we aimed to identify the genetic features in a family with lethal respiratory failure.

**Methods:**

We studied members of two generations of a Chinese family, including a female proband, her parents, her monozygotic twin sister, and her older sister. Trio whole exome sequencing (WES) were used on the proband and her parents to identify the *ABCA3* variants. Sanger sequencing and real-time quantitative polymerase chain reaction (PCR) were used on the monozygotic twin sister of proband to validate the *ABCA3* synonymous variant and exon deletion, respectively. The potential pathogenicity of the identified synonymous variant was predicted using the splice site algorithms dbscSNV11_AdaBoost, dbscSNV11_RandomForest, and Human Splicing Finder (HSF).

**Results:**

All patients showed severe respiratory distress, which could not be relieved by mechanical ventilation, supplementation of surfactant, or steroid therapy, and died at an early age. WES analysis revealed that the proband had compound heterozygous *ABCA3* variants, including a novel synonymous variant c.G873A (p.Lys291Lys) in exon 8 inherited from the mother, and a heterozygous deletion of exons 4–7 inherited from the father. The synonymous variant was consistently predicted to be a cryptic splice donor site that may lead to aberrant splicing of the pre-mRNA by three different splice site algorithms. The deletion of exons 4–7 of the *ABCA3* gene was determined to be a likely pathogenic variant. The variants were confirmed in the monozygotic twin sister of proband by Sanger sequencing and qPCR respectively. The older sister of proband was not available to determine if she also carried both *ABCA3* variants, but it is highly likely based on her clinical course.

**Conclusions:**

We identified a novel synonymous variant and a deletion in the *ABCA3* gene that may be responsible for the pathogenesis in patients in this family. These results add to the known mutational spectrum of the *ABCA3* gene. The study of *ABCA3* variants may be helpful for the implementation of patient-specific therapies.

**Supplementary Information:**

The online version contains supplementary material available at 10.1186/s12920-021-01098-4.

## Background

Neonatal hyaline membrane disease, neonatal transient tachypnoea, meconium aspiration syndrome, and infectious pneumonia are common causes of neonatal respiratory distress. Primary or secondary pulmonary surfactant deficiency causes fatal respiratory failure, the main cause of neonatal death among preterm infants. Surfactant deficiency is typically associated with a developmental insufficiency due to prematurity. When a full-term newborn has persistent clinical and X-ray manifestations of respiratory distress syndrome (RDS), and the response to supplemental exogenous pulmonary surfactant therapy is transient, the possibility of a rare inherited pulmonary surfactant deficiency should be considered. The surfactant present in the lungs of all mammals is a complex compound of phospholipids and proteins. It maintains effective ventilation in the lungs by reducing the surface tension in the alveoli [1]. ABCA3 (The adenosine triphosphate (ATP) binding cassette subfamily A, member 3) hydrolyzes ATP to transport phospholipids which combine with surfactant proteins to yield pulmonary surfactant [2, 3].

Surfactant metabolism disorders caused by genetic variants are a group of diseases with a wide range of clinical manifestations, ranging from fatal respiratory distress in newborns to interstitial lung disease in children or adults [4, 5]. Variants in the *ABCA3* (OMIM acc, No. 601615), *SFTPB* (OMIM acc, No. 178640), and *SFTPC* (OMIM acc, No. 178620) genes can cause qualitative and quantitative defects in surfactant [5]. However, genetic surfactant deficiency is mainly caused by biallelic variants in *ABCA3*, which is located on chromosome 16p13.3 [6]. Exonic deletions involving *ABCA3* are rare [7–9], and a disease caused by synonymous variants has only been reported last year [10]. In this study, we reported the clinical and genetic characteristics of a Chinese family, including three sisters who were born with fatal respiratory failure.

## Methods

### Clinical specimens

This study investigated members of two generations of a Chinese family, containing female proband, her sisters, and her parents, from the Quanzhou Women and Children’s Hospital. This study was approved by the ethics committee of Quanzhou Women and Children’s Hospital and written consent was obtained from the guardians of all participants.

### DNA extraction and Sanger sequencing

Genomic DNA was extracted from peripheral whole blood or dried blood spots obtained from the proband, her parents, and her monozygotic twin sister. We could not obtain the genomic DNA of the proband’s older sister, because she died early. All DNA was extracted from peripheral blood white blood cells. The coding region and flanking intron sequences of the *ABCA3* (NM_001089) gene were amplified using standard polymerase chain reaction (PCR) conditions and bi-directional DNA sequencing. The DNA from the proband, her parents, and her monozygotic twin sister was analysed using Sanger sequencing for the *ABCA3* variant c.G873A (p.Lys291Lys). DNA sequences, including the candidate variant, were amplified using the forward primer *ABCA3*-F:5′-AAGTCACTCTGTTGCCCCAA-3′ and the reverse primer *ABCA3*-R:5′ -CACCTATAGTCCCAACTACTC-3′. SuperReal PreMix Plus (SYBR Green) (FP205, Tiangen Biochemical Technology Co., Ltd., Beijing, China) was used. The PCR cycle included the following steps: 2 min at 95 °C, followed by 36 cycles of 30 s at 95 °C, 1 min at 60 °C, 1 min at 72 °C, and a final step at 72 °C for 2 min.

### Whole exome sequencing

Whole exome sequencing (WES) was performed to identify any underlying pathogenic variants in the proband and her parents. First, Blood Genome Extraction Kits (Tiangen Biochemical Technology Co., Ltd.) were used to extract the genomic DNA from the leucocytes in the blood sample. DNA was sheared with the Bioruptor Pico Sonication System. NEBNext Ultra II DNA Library Prep Kits (New England Biolabs, Ipswich, MA, USA) were used for library preparation. The samples were submitted for 150 bp pair-end sequencing, performed on a NextSeq 500 Sequencing System (Illumina, San Diego, USA) by the Genokon Medical Laboratory, Xiamen, China. Trimmomatic (Usadel Lab, Aachen, Germany) was used to perform quality control and remove data of low quality. The Burrows-Wheeler Alignment tool (BWA) [11] was used to align reads to the reference (GRCh37/hg19). Variant discovery and genotyping were performed with GATK (https://software.broadinstitute.org/gatk/) and annotated with ANNOVAR [12]. Common variants, such as intergenic, upstream, downstream, intronic, and synonymous variants, and variants with minor allele frequency (MAF) > 1% in the 1,000 genome, ExAC, and gnomAD databases, were filtered out. In silico programs were used to predict the deleterious effect of each variant on the function of the proteins, including REVEL [13], ClinPred [14], SIFT [15], Polyphen2 [16], LRT [17], MutationAssessor [18], PROVEAN [19], CADD [20], MutationTaster [21], dbscsnv11_AdaBoost [22], dbscsnv11_RandomForest [22], and Human Splicing Finder (HSF) [23]. Genotype–phenotype analyses were performed using the Exomiser [24] and Phenolyzer [25] software programs. Finally, we read the results according to the standards and guidelines of American College of Medical Genetics and Genomics (ACMG) [26].

### Real-time quantitative PCR

The deletions from exons 4 to 7 of the *ABCA3* gene of the monozygotic twin sister of proband were validated using real-time quantitative PCR as described above. All reactions were performed in triplicate. Primer sequences are presented in Additional file 1.

## Results

### Clinical information

**Fig. 1:**
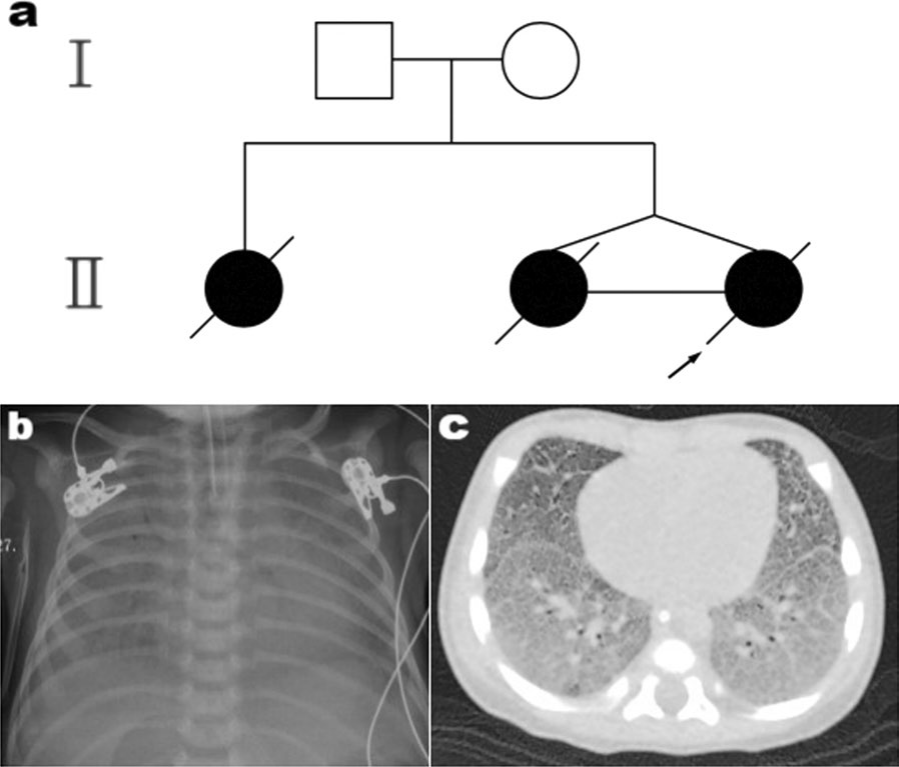
Clinical information about the family. **a** Pedigree of the family. The arrow denotes the proband and the filled black symbols represent the affected members. **b** X-rays of the proband revealed the bilateral "white lungs" at six days of admission. **c** The chest computed tomography of the older sister showed interstitial lung disease at 56 days

The patients were from a family in Quanzhou, Fujian province, China (Fig. 1a). The proband and her monozygotic twin sister were born without complications by caesarean delivery at 38 weeks and three days, with Apgar scores of 10 at one, five, and ten min. The birth weight of the proband and her sister was 2650 g and 2300 g, 14th and 3rd percentile of birth weight on Fenton growth chart [27], respectively. Their mother had no fever, no infection, and no intrauterine hypoxia in late pregnancy. After birth, the proband suffered from shortness of breath, grunting, and cyanosis, and was transferred to the neonatal intensive care unit (NICU). The proband was treated with non-invasive positive pressure ventilation, invasive mechanical ventilation, and high frequency oscillation ventilation. She was also treated with a combination of pulmonary surfactant supplementation, antibiotics, inhalation of nitric oxide (NO) to reduce persistent pulmonary hypertension, and the anti-inflammatory methylprednisolone. The echocardiogram did not reveal any structural abnormalities of the heart. The chest radiograph of the proband at birth showed reticular granular blur of both lungs, and then progressed to bilateral "white lungs" (Fig. 1b) at six days of admission. Despite these treatments, the proband’s condition worsened rapidly, presenting with refractory dyspnoea, hypoxemia, persistent pulmonary hypertension, and eventually leading to death at the age of 23 days.

The monozygotic twin sister of the proband also suffered from shortness of breath, grunting, and cyanosis after birth. In the neonatal intensive care unit, treatment with non-invasive positive pressure ventilation, invasive mechanical ventilation, high frequency oscillation ventilation, antibiotics, glucocorticoids, pulmonary surfactant supplementation and inhaled nitric oxide were also performed. She died in 23 days after birth due to refractory dyspnoea, hypoxemia, and persistent pulmonary hypertension.

Inquiring about family history revealed that the proband had an older sister born from her mother’s first pregnancy more than a year earlier. The older sister was delivered by vaginal delivery at 38 weeks and 6 days, after an uncomplicated pregnancy, with an Apgar score of 10 at one, five and ten min, and birth weight of 3200 g, 49th percentile of birth weight on Fenton growth chart [27]. Her condition was similar to that of the proband. However, she had a relatively long clinical treatment process. Fifteen days after birth, the patient's breathing improved after supplementation with pulmonary surfactant, and she was out of oxygen therapy for eight days. However, she still showed symptoms of respiratory failure, such as dyspnea, cyanosis, and, and hypoxemia, and she was unable to do without oxygen support until her death. From 35 to 42 days after birth, she was continuously treated with methylprednisolone and azithromycin, but no significant improvement was observed. Chest CT at 56 days showed interstitial lung disease (Fig. 1c). She died 109 days after birth due to refractory dyspnoea and hypoxemia.

The clinical presentations and suspicious family history led us to hypothesise that there was a genetic cause. Peripheral blood samples were obtained from the infants and parents for WES and further genetic analysis. However, we were unable to obtain pathological specimens because the parents refused fiberoptic bronchoscopy, lung biopsy, and autopsy.

### Sequencing and qPCR results: *ABCA3* variants identified by WES

**Fig. 2:**
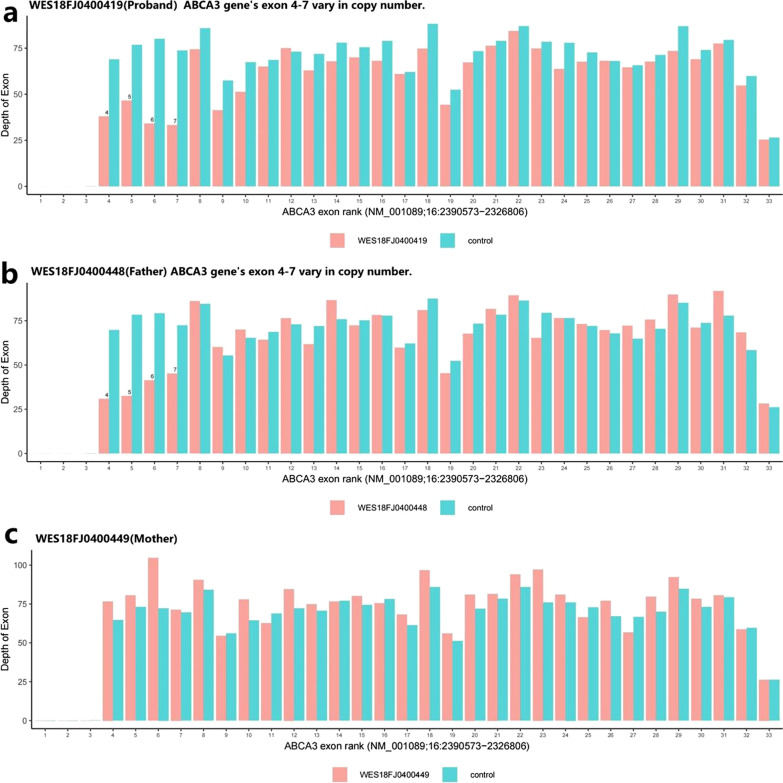
*ABCA3* variants in this family. **a**, **b** Results of whole exome sequencing (WES) showed deletion of exons 4–7 of the *ABCA3* gene in the samples of the proband and their father. **c** Deletion of exons 4–7 of the *ABCA3* gene were not found in the samples from the proband’s mother

**Fig. 3:**
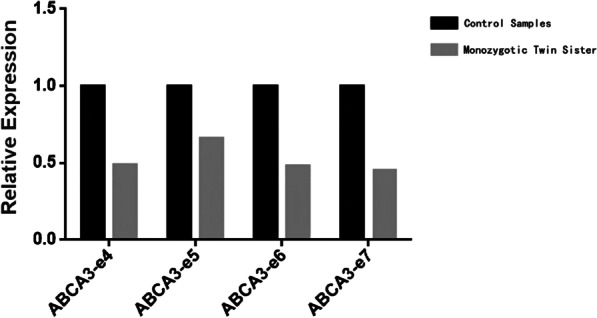
*ABCA3* variant in the monozygotic twin sister of the proband. Copy number variations analysis of exons 4–7 of *ABCA3* by qPCR. The monozygotic twin sister of proband, has a value around 0.5 of relative expression, comparing to the control samples (value = 1.0), indicates heterozygous deletion of exons 4–7 of *ABCA3*

Two *ABCA3* variants were identified by WES of the proband’s DNA sample, specifically a heterozygous deletion of exons 4–7 (Fig. 2), and a novel heterozygous synonymous variant c.G873A (p.Lys291Lys) in exon 8. The *ABCA3* gene variants were subsequently determined to be *in trans* in the proband, with the unaffected father found to be carrying the heterozygous deletion of exons 4–7 and the unaffected mother carrying the heterozygous synonymous variant c.G873A (p.Lys291Lys) (Table 1). We further confirmed the *ABCA3* variants in a DNA sample from the proband’s monozygotic twin sister. The result of real-time quantitative PCR experiment revealed a heterozygous deletion of exons 4–7 (Fig. 3). The heterozygous c.G873A (p.Lys291Lys) variant of the *ABCA3* gene was identified by Sanger sequencing. These results were consistent with those of the proband.

**Table 1:** *ABCA3* variants identified

Gene	Chromosome	Nucleotide Change	Protein Change	Variant type	In silico prediction	Genotype	Parent of origin
*ABCA3*	Chr16:2390573−2326806	NM_001089:(Chr16:2390573−2326806)X1	Exons 4–7 deletion	Deletion	–	Heterozygous	Paternal
*ABCA3*	Chr16:2369582	NM_001089: exon8:c.873G>A	p.Lys291Lys	Synonymous	Cryptic splice site activation	Heterozygous	Maternal

The deletion of exons 4–7 of the *ABCA3* gene was identified by bioinformatic analysis of single-gene copy number variants using NGS data. This deletion was absent from the gnomAD and ExAC databases. It was interpreted as likely pathogenic according to the ACMG guidelines (PVS1_Strong + PM2_Supporting + PP1) [26]. The c.G873A (p.Lys291Lys) novel variant was synonymous, however, it was also absent from the gnomAD, HGMD, 1,000 Genomes, and EXAC databases. It was located one base pair from an exon–intron junction, a donor site, and was consistently predicted to be a novel cryptic splice donor site by three different splice site algorithms (dbscSNV11_AdaBoost, dbscSNV11_RandomForest, and HSF) within exon 8 of the *ABCA3* gene, which may lead to aberrant splicing of the pre-mRNA (see Additional file 2). Given the consistent in silico prediction of a cryptic splice site, the absence in large population sequencing database, and presence *in trans* with the likely pathogenic variant, deletion of exons 4–7, in two patients, both the proband and her monozygotic twin sister, this variant was also interpreted as likely pathogenic according to the ACMG guidelines (PM2_supporing + PP3 + PM3_strong + PP1). Therefore, the evidence supported the diagnosis of autosomal recessive pulmonary surfactant metabolism dysfunction caused by deficiency of *ABCA3*.

## Discussion

Human respiration depends on the extensive gas exchange surface area provided by the expanded alveoli. Lipid-rich lung surfactant keep the alveoli unobstructed throughout the air–liquid interface. The synthesis, transport, secretion, and recycling of surfactant occurs in alveolar epithelial type II cells (AEC2). Alveolar macrophages also participate in the recycling of surfactant [28]. ABCA3, a transmembrane phospholipid glycoprotein, is a member of the ABC ATP binding cassette family that is essential for the formation of lamellar bodies (LBs) and phospholipid transport, as well as assembly and generation of surfactant [29, 30]. *ABCA3* is expressed in a number of tissues, the only disease associated with biallelic variants is lung disease. *ABCA3* variants may affect a range of physiological processes in lung. *ABCA3* variants identified in patients have been modelled in vitro in cell-based systems including A549 and HEK293 cells [31, 32]. These mechanisms include altered intracellular trafficking of ABCA3, impaired ATP hydrolysis-mediated phospholipid transport, or promotion of a toxic gain-of-function phenotype through the induction of cell stress pathways [33–36]. The most common ABCA3 variant, p.Glu292Val, has been shown to result in impaired ATP hydrolysis-mediated phospholipid transport [2, 34, 37]. In our study, the novel variant c.G873A: p.Lys291Lys is located very close to the p.Glu292Val. However, the mechanisms of pathogenesis of p.Lys291Lys have not been functionally characterized. 

Fatal RDS caused by biallelic variants in *ABCA3* among newborns with congenital surfactant deficiency was first reported in 2004 [38]. So far, more than 200 *ABCA3* variants have been found, and about three-quarters of patients present with compound heterozygosity [24]. At present, the incidence of *ABCA3* variants in newborns is not clear. Wambach et al. predicted that the disease incidence of the *ABCA3* variant ranged from 1:4000 to 1:17,000 in individuals of European and African descent, but this is likely an overestimate as not all missense variants are pathogenic and fewer babies with ABCA3 deficiency are identified each year than would be predicted [39]. A previous study showed that *ABCA3* is the most frequent genetic variant affecting the function of surfactant, at 2.7%, in the mixed ethnic Han and Zhuang populations in Nanning, China [40].

Although uniparental disomy has been reported, the most common pattern of *ABCA3* variants is autosomal recessive, requiring variants in both alleles [7]. Kroner et al. studied 242 patients with interstitial lung disease (ILD) by *ABCA3* gene sequencing, and found that 69 patients had at least one variant, and 40 of the 69 patients had two different pathogenic variants [41].

In this study, we identified heterozygous deletion variants in exons 4–7 of *ABCA3* gene in both proband and paternal samples using WES. According to the qPCR results, a heterozygous deletion variant of exons 4–7 of *ABCA3* gene was also present in the monozygotic twin sister of the proband. There have been few reports on exon deletions in *ABCA3*. A large homozygous deletion variant in exons 2–5, a heterozygous deletion variant in exons 13–18, and a heterozygous deletion variant in exon 12 have been reported to cause neonatal respiratory distress [7–9]. In animal experiments, a mouse model of the deletion of *ABCA3* exons 4–7 showed the mechanism of the *ABCA3* deletion leading to respiratory failure in mice [29, 42]. Through WES, the *ABCA3* gene c.G873A: p.Lys291Lys heterozygous variant was identified in the samples of the proband and their mother, and the same results were found in the monozygotic twin sister by Sanger sequencing. The heterozygous variant of the *ABCA3* gene C.G873A is synonymous, predicted to be a novel cryptic splice donor site by three different splice site algorithms, and is absent from the gnomAD, HGMD, 1000 Genomes and EXAC gene databases. In 2014, Wambach et al. reported that common synonymous *ABCA3* variants were not overrepresented among term newborn RDS patients [43]. Oltvai et al. first reported a patient with an *ABCA3* synonymous variant. The clinical manifestations and weak *ABCA3* immunostaining provided evidence that the c.2883C>T p.Gly961Gly variant behaved like a "mosaic null" allele. The *ABCA3* synonymous variant c.2883C>T p.Gly961Gly is predicted to alter RNA splicing and may be pathogenic [10]. This is the second time that this synonymous variant has been reported as a cryptic exonic splicing variant in *ABCA3* though it has not yet been validated on RNA level. NGS sequencing did not find other variants affecting surfactant, including genes such as *SFTPB*, *SFTPC*, *NKX2-1*, and *FOXF1*. The combination of clinical phenotypes, algorithm predictions, and genetic findings supports our theory that these variants are pathogenic. There is a limitation that DNA from the older sister of proband was not available to determine if she also carried both *ABCA3* variants, but it is highly likely based on her clinical course.

There is a recognized correlation between genotype and phenotype in *ABCA3* deficiency. Patients with two null variants, caused by a premature stop codon or frame shifts, have earlier symptoms and greater mortality in the first year of life than other patients with *ABCA3* deficiency due to missense variants [44]. It is generally believed that besides lung transplantation, patients with *ABCA3* gene variants have no effective treatments, although the combination of corticosteroids, macrolides, and hydroxychloroquine has been used in clinical empirical therapy. However, studies have shown that exogenous surfactant, whole-lung lavage, hydroxychloroquine, and corticosteroids have multiple significant but transient effects on individuals [41, 44]. Blinded controlled treatment evaluations for rare diseases have not yet proven feasible [36]. Our patients had a fatal early-onset disease with serious progression, needing long-term respiratory support and oxygen supplementation. Even the repeated use of surfactant, macrolides, and corticosteroids did not lead to significant clinical improvement. All three patients eventually died at an early stage in life.

This study has a significant limitation should be noted. Due to lung tissue from the three infants was not available and *ABCA3* is not sufficiently expressed in the peripheral blood, demonstrating the synonymous *ABCA3* variant alters splicing would be difficult to complete. However, given that highly identical clinical manifestations and genetic findings in these patients, we speculate that the synonymous variant and deletion in *ABCA3* may be responsible for the pathogenesis in patients in this family. Further functional studies are warranted to confirm the pathogenicity of the synonymous variant.

## Conclusions

In conclusion, we reported the clinical and genetic features of a Chinese family with compound heterozygous variant in the *ABCA3* gene: a novel variant c.G873A:p.Lys291Lys and the deletion of exons 4–7. Reports on novel *ABCA3* gene variants, clinical course, and treatment response, especially *ABCA3* gene synonymous variants that cause the disease, will help further understand the diagnosis and develop treatment strategies for *ABCA3* deficiency.

## Supplementary Information

**Additional file 1: Table S1.** Primer sequences used in exons 4 to 7 of *ABCA3* gene amplification.

**Additional file 2: Table S2.** *In silico* prediction of the *ABCA3* synonymous variant c.873G > A.

## Data Availability

The datasets generated during and/or analysed during the current study are available in the NCBI BioProject repository (https://www.ncbi.nlm.nih.gov/bioproject/PRJNA639422).

## Abbreviations

WES: Whole exome sequencing
PCR: Polymerase chain reaction
RDS: Respiratory distress syndrome
ATP: Adenosine triphosphate
CT: Computerized tomography
