# Supplementary material for: A novel synonymous ABCA3 variant identified in a Chinese family with lethal neonatal respiratory failure
Source: BMC Med Genomics. 2021 Oct 29;14:256. doi: 10.1186/s12920-021-01098-4 (PMC8556997; doi:10.1186/s12920-021-01098-4)
Supplement: Supplementary file 1 — Additional file 1: Table S1. Primer sequences used in exons 4 to 7 of ABCA3 gene amplification. [file 12920_2021_1098_MOESM1_ESM.doc]

**Table S1.** Primer sequences used in exons 4 to 7 of *ABCA3* gene amplification

| Exons | forward | Reverse |
| --- | --- | --- |
| *ABCA3*-e4 | 5’-CCCCACTCTGCGTGTTTC-3’ | 5’-TGCCTGAGCACAGCCATC-3’ |
| *ABCA3*-e5 | 5’-AGTCGGAAAATGTGCCCAAC-3’ | 5’-AAGGGATGTAGGCAAGCTCC-3’ |
| *ABCA3*-e6 | 5’-TCTGCCAGTGACCTGAACC-3’ | 5’-ACGCTGGACGAGCAGTTGT-3’ |
| *ABCA3*-e7 | 5’-TATCACCTACGGTTCAGTTACAC-3’ | 5’-CCTTGGTCCTGGGTTTGG-3’ |
| *ALB* | 5’-TCTGCTCTCCTGCCTGTTCT-3’ | 5’-TCGCCTGTTCACCAAGGAT-3’ |
