# Supplementary material for: A novel synonymous ABCA3 variant identified in a Chinese family with lethal neonatal respiratory failure
Source: BMC Med Genomics. 2021 Oct 29;14:256. doi: 10.1186/s12920-021-01098-4 (PMC8556997; doi:10.1186/s12920-021-01098-4)
Supplement: Supplementary file 2 — Additional file 2: Table S2. In silico prediction of the ABCA3 synonymous variant c.873G > A. [file 12920_2021_1098_MOESM2_ESM.doc]

**Table S2.** *In silico* prediction of the *ABCA3* synonymous variant c.873G>A

| **Software** | **Score** | **Interpretation** |
| --- | --- | --- |
| AdaBoost^a^ | 1 | splice-altering |
| Random Forest^a^ | 0.976 | splice-altering |
| HSF^b^ | -10.08  (87>76.92=>-11.59%) | Alteration of the WT donor site, most probably affecting splicing. |

^a^AdaBoost and Random Forest: A score of more than 0.6 is predicted as splice-altering [1].

^b^HSF: A score of less than 0 is considered to be an altered splice site [2]. (https://hsf.genomnis.com/)

**References**

1. Jian X, Boerwinkle E, Liu X: **In silico prediction of splice-altering single nucleotide variants in the human genome**. *Nucleic Acids Res* 2014, **42**(22):13534-13544.

2. Desmet FO, Hamroun D, Lalande M, Collod-Béroud G, Claustres M, Béroud C: **Human Splicing Finder: an online bioinformatics tool to predict splicing signals**. *Nucleic Acids Res* 2009, **37**(9):e67.
